# Elevated Matrix Metalloproteinase Type 9 (MMP-9) Transcripts After Thymoglobulin Induction in Incident Kidney Transplant Recipients

**DOI:** 10.3390/ijms26199310

**Published:** 2025-09-24

**Authors:** Tor B. B. Petersen, Subagini Nagarajah, Martin Tepel

**Affiliations:** 1 Department of Clinical Research, Department of Molecular Medicine, University of Southern Denmark, Campusvej 55, 5230 Odense, Denmark; torbbpetersen@hotmail.com; 2 Department of Nephrology, Odense University Hospital, Kløvervænget 2 D, 5000 Odense, Denmark

**Keywords:** renal transplant, outcome, transcripts, matrix metalloproteinase, delayed allograft function

## Abstract

Matrix metalloproteinase type 9 (MMP-9), which cleaves collagen type IV in basal membranes, has been associated with the progression of chronic kidney disease. The objective of the present study was to evaluate the characteristics of donors, recipients, induction therapies, and allograft function on MMP-9 transcripts from mononuclear cells in kidney transplant recipients. Transcripts were determined in peripheral blood mononuclear cells from 67 incident renal transplant recipients eight days post-transplant using quantitative real-time PCR and quantified using the ΔΔCq method. Median MMP-9 transcripts were 6.1 (IQR, 1.5 to 66.5, N = 4) in AB0-incompatible donor transplants; 3.2 (IQR, 2.0 to 16.9, N = 17) in living donor transplants; and 4.2 (IQR, 2.3 to 9.2, N = 46) in deceased donor transplants (*p* = 0.8). Importantly, renal transplant recipients who were treated with thymoglobulin had significantly higher median MMP-9 transcripts compared to all other induction therapies (14.5; IQR, 2.8 to 31.9, N = 10; vs. 3.5, IQR, 2.2 to 8.8, N = 57; *p* = 0.01). Median MMP-9 transcript levels were similar in recipients with delayed allograft function and immediate allograft function (8.86; IQR, 5.29 to 11.57, N = 7; vs. 3.4; IQR, 2.35 to 9.49, N = 60; *p* = 0.245). Induction therapy with thymoglobulin causes significantly higher MMP-9 transcripts in peripheral blood mononuclear cells, probably indicating an increased inflammatory response.

## 1. Introduction

Kidney transplantation is the treatment of choice for patients with end-stage renal disease in terms of quality of life, survival, and cost-effectiveness [1,2,3,4]. However, there is a global shortage of donor kidneys [5]; hence, efforts should be made to prolong kidney transplant survival to reduce the need for re-transplant [6,7]. Biomarkers may help to predict long-time kidney transplant survival [8]. For example, the extracellular matrix gelatinase MMP-9, which cleaves collagen type IV in basal membranes, has been associated with inflammation and the progression of chronic kidney disease [9]. Chronic allograft nephropathy, a leading cause of kidney allograft loss, is characterized by excessive accumulation of the extracellular matrix [10]. As reported by Tan and Liu (2012), the extracellular matrix gelatinase MMP-9 may largely contribute to extracellular matrix accumulation, which finally leads to loss of the allograft function [11]. MMP-9 belongs to the family of zinc-dependent endopeptidases, which are able to specifically cleave extracellular matrix, i.e., degradation of type-IV collagen, laminin, and fibronectin [12]. MMP-9 promotes inflammation and is involved in angiogenesis and wound healing [9,13]. MMP-9 is expressed in the kidney, i.e., in glomerular, mesangial, epithelial, endothelial, and collecting duct cells, as well as in mononuclear cells [14,15]. Akbari et al. reported the gene expression of MMP-9, interleukon-6, and tumor necrosis factor alpha in peripheral blood mononuclear cells [15]. Mononuclear cells are involved in the immune response after kidney transplantation. Mononuclear cells may recognize the transplanted allograft as foreign and activate an inflammatory response. That inflammatory response may damage the allograft and reduce its function. Immunosuppressive drugs including thymoglobulin reduce that inflammatory response and help maintain the non-self allograft. Thymoglobulin contains antibodies against markers on the T-cell surface, for example, CD4, CD8, CD11a, CD25, and CD44. Thymoglobulin reduces mononuclear cells via apoptosis and cytotoxic mediators. It is currently unknown whether donor and recipient characteristics as well as induction therapies may influence transcripts in mononuclear cells and accordingly the course after kidney transplantation. Mononuclear cells are involved in the immune response after kidney transplantation.

The objective of the present study was to determine MMP-9 transcripts in mononuclear cells from kidney transplant recipients as an essential marker of the inflammatory response. MMP-9 was determined after deceased as well as living donor transplantation. We observed higher MMP-9 transcripts in peripheral blood monocytes from incident kidney transplant recipients who were treated with thymoglobulin induction therapy.

## 2. Results

### 2.1. Patient Characteristics

Matrix metalloproteinase 9 (MMP-9) transcript levels were measured in mononuclear cells from 67 incident kidney transplant recipients (KTRs). In detail, 46 KTRs were male (69%) and 21 were female (31%). The median age of KTRs at the time of transplantation was 55 years (IQR, 45.5 to 62.5 years). Most of KTRs had end-stage renal disease due to glomerulonephritis (40%). The clinical characteristics of all 67 KTRs are presented in Table 1 and Table 2.

Further, 4 recipients (6%) obtained AB0-incompatible donor kidney transplants, 17 recipients (25%) obtained AB0-compatible living donor kidney transplants, and 46 recipients (68%) obtained deceased donor transplants. Also, 10 recipients received thymoglobulin as induction therapy, 57 recipients received other induction therapies, 7 recipients experienced delayed graft function, whereas 60 had immediate allograft function.

### 2.2. MMP-9 Transcripts in Blood Mononuclear Cells from Recipients, Who Obtained a Transplant from AB0-Incompatible Living Donors, AB0-Compatible Living Donors and Deceased Donors, 8 Days After Transplantation

**Figure 1:**
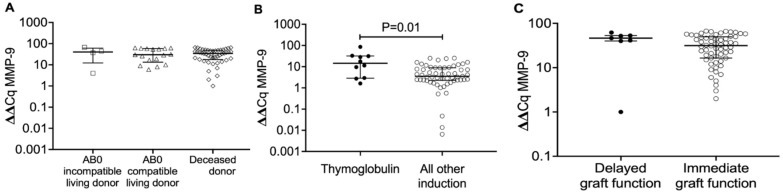
Scatterplots displaying ΔΔCq MMP-9 levels in peripheral blood mononuclear cells taken from a total of 67 kidney transplant recipients 8 days after kidney transplantation. (**A**) Kidney transplant recipients stratified into either AB0-incompatible living donor (N = 4), AB0-compatible living donor (N = 17) or deceased donor (N = 46). Median and interquartile range (IQR) is indicated. Difference between groups is compared by Kruskal–Wallis test, *p* = 0.8. (**B**) Kidney transplant recipients stratified into either thymoglobulin (N = 10) or all other induction therapies (N = 57). Median and interquartile range (IQR) are indicated. Difference between groups is compared with Mann–Whitney test, *p* = 0.01. (**C**) Kidney transplant recipients stratified into either delayed graft function (N = 7) or immediate graft function (N = 60). Median and interquartile range (IQR) are indicated. Difference between groups is compared with Mann–Whitney test, *p* = 0.245.

Median MMP-9 transcripts in kidney transplant recipients were 6.1 (IQR, 1.5 to 66.5) in AB0-incompatible living donors, N = 4, vs. median 3.2 (IQR, 2 to 16.9) in AB0-compatible living donors, N = 17, vs. 4.2 (IQR, 2.3 to 9.2) in deceased donor transplants, N = 46 (*p* = 0.8 in Kruskal–Wallis test), revealing no statistically significant difference between groups. The distribution of MMP-9 transcripts according to donor characteristics is shown in a scatterplot in Figure 1A.

### 2.3. MMP-9 Transcripts in Blood Mononuclear Cells from Kidney Transplant Recipients According to Type of Induction Therapy (Thymoglobulin vs. All Other Induction Therapies) 8 Days After Transplantation

A total of 10 KTRs received thymoglobulin induction therapy (15%), 56 recipients received Basiliximab (83.6%), 4 recipients received rituximab (6%), and 22 recipients received prednisolone (32.8%). Note that recipients could receive multiple modalities of induction therapy at the same time, explaining why the induction therapy percentage adds up to more than 100 percent. The clinical characteristics of KTRs receiving either thymoglobulin or all other induction therapies are given in Table 2.

As shown in Table 2, the clinical and laboratory characteristics were similar in the thymoglobulin and the all-other-induction-therapies group, with the exception of glomerulonephritis as an underlying cause of end-stage chronic kidney disease. That is due to the fact that according to local guidelines, all patients with glomerulonephritis were treated with thymoglobulin [16].

It should be noted that kidney transplant recipients who were treated with thymoglobulin had statistically significantly higher MMP-9 transcript levels in their remaining mononuclear cells compared to all other induction therapies (median 14.5; IQR, 2.8 to 31.9; N = 10; vs. median, 3.5, IQR, 2.2 to 8.8, N = 57; *p* = 0.01 by Mann–Whitney test). The distribution of MMP-9 transcripts according to induction therapy is shown in a scatterplot in Figure 1B.

### 2.4. MMP-9 Transcript Levels in Blood Mononuclear Cells from Kidney Transplant Recipients 8 Days After Kidney Transplantation in Delayed Versus Immediate Graft Function

Kidney transplant recipients with immediate and those with delayed allograft function did not show a significantly different MMP-9 transcript level (median 8.86; IQR, 5.29 to 11.57; N = 7; versus median 3.40; IQR, 2.35 to 9.49; N = 60; Mann–Whitney test *p* = 0.245). The distribution of MMP-9 transcripts according to delayed vs. immediate graft function is shown in a scatterplot in Figure 1C.

## 3. Discussion

Our present investigation showed that compared to other induction therapies, kidney transplant recipients (KTRs) treated with thymoglobulin have significantly higher MMP-9 transcripts in peripheral blood mononuclear cells. Since MMP-9 has been associated with extracellular matrix accumulation, angiogenesis, wound healing, and collagen degradation [9], higher MMP-9 transcript levels eight days after kidney transplant may indicate an increased inflammatory response in mononuclear cells, which remain after the administration of thymoglobulin, which depletes mononuclear cells. Homeostatic repopulation of T-lymphocytes, NK cells and monocytes after thymoglobulin administration may be the underlying cause for the observed effects on transcripts.

MMPs contribute to inflammatory cell migration and to increased vascular permeability. MMP-9 levels rise in experimental renal ischemia and may promote neutrophil extravasation by degrading ECM during reperfusion injury.

It may be hypothesized that a type III immune reaction causing an inflammatory response also occurred in our group after xenogentic protein administration. Therefore, it may be plausible that the observed upregulation of MMP-9 expression reflects a reaction to xenogentic protein. That may be the underlying cause for increased MMP-9 transcripts rather than the induction therapy per se.

Inhibition of MMP-9 has been shown to protect against renal reperfusion injury in experimental models, and MMP-9 is also associated with acute renal allograft rejection [17]. Given the role of MMP-9 in inflammation and ischemia–reperfusion injury, donor-related factors may influence its expression. Donor characteristics such as AB0 blood group compatibility and deceased donor status are known to affect allograft outcomes [18]. KTRs received immediate induction therapy before transplant and maintenance immunosuppression therapy, which is given as long as the allograft persists [19]. The goal of induction therapy is to suppress KTRs’ T-cell response to allograft antigen presentation. This is achieved using either biologic lymphocyte-depleting agents, such as anti-thymocyte globulin (Thymoglobulin), or interleukin-2 receptor antagonists including Basiliximab [19]. Thymoglobulin is composed of purified polyclonal rabbit-derived immunoglobulins targeting human thymocytes, acting by depleting T cells in both circulation and secondary lymphatic tissues, rapidly after administration [16].

Our findings are in line with findings from Turunen et al., who measured systemic MMP-9 levels obtained from central venous blood samples preoperatively before kidney transplantation, before reperfusion, as well as after reperfusion [17]. Turunen et al. reported higher MMP-9 levels in kidney transplant recipients who received thymoglobulin after transplant [17]. It is known that peripheral blood mononuclear cells, which are isolated from peripheral blood, contain several cell types, including lymphocytes, monocytes, natural killer cells or dendritic cells. Thymoglobulin mainly depletes CD3+ T cells. Hence, our findings may indicate that the amounts of MMP-9 transcripts may either be low in CD3+ T cells and/or reactive stimulation of MMP-9 transcripts in other peripheral blood mononuclear cells. Future studies in several peripheral blood mononuclear subtypes may be necessary to quantify MMP-9 findings in distinct subtypes. Currently, it is unknown whether the increased MMP-9 transcript level after thymoglobulin is also present on either the protein MMP-9 protein level or the degree of cell activation.

We also investigated whether the MMP-9 transcripts affect the immediate allograft function post-transplant. Delayed graft function (DGF) is defined as the need for dialysis within one week after kidney transplantation for any reason [20]. Delayed allograft function is associated with higher morbidity and healthcare costs [20]. We observed that MMP-9 transcript levels were similar in patients with delayed graft function and immediate graft function. In contrast, Zhao et al. observed an increased serum MMP-9 level three days post-transplant in AB0-compatible living donor transplant recipients who experienced acute allograft rejection [21]. Kamińska et al. reported an increased donor expression of MMP-9 in 6 out of 33 recipients who showed delayed graft function after deceased donor kidney transplant (*p* = 0.04) [22]. Hence, it appears that induction therapy may have larger effects on the expression of MMP-9 rather than the immediate excretory function of the allograft. There may be possible limitations in our study due to limited sample size for the donor subgroup, i.e., AB0-incompatible lining donors. Further studies may be necessary with larger sample sizes and determinations of protein abundance as well. A limitation of our study may be that it was restricted to one inflammatory transcript. Future studies with additional inflammatory markers may be necessary to confirm that induction therapies affect various transcript levels in peripheral blood mononuclear cells. It may be assumed that cytokine transcripts, for example, interleukin 10 or tumor necrosis factor, may be affected as well. Furthermore, the examination of the time course of transcripts before and immediately after, as well as at greater intervals, weeks or months, after transplantation is necessary to obtain a comprehensive picture of transcripts in peripheral blood cells.

## 4. Materials and Methods

### 4.1. Ethical Statement

The study protocol was in accordance with the ethical standards of the Declaration of Helsinki and in the Declaration of Istanbul on Organ Trafficking and Transplant Tourism. This study was approved by the local ethics committee (Den Videnskabsetiske Komite for Region Syddanmark, Projekt-ID: 20100098). Written informed consent was obtained from all patients before entry into the study. The consent form for participation was distributed to all participants and signed. Exclusion criteria were age below 18 years or missing consent.

### 4.2. Study Cohort

Baseline characteristics of donors and recipients and information on organ procurement were prospectively obtained from medical records. Induction therapy, immunosuppressive therapy, concomitant medications were all made by the clinicians at the institution according to the local protocol. Physicians were unaware of the transcript levels.

Immunosuppressive induction therapies included interleukin 2-receptor antibodies (Basiliximab), anti-thymocyte globulins (Thymoglobulin, 1.5 mg/kg daily for 4 days), prednisolone, and anti-CD20 antibodies (Rituximab). Immunosuppressive maintenance therapies included interleukin 2 antagonists (tacrolimus) and inosine monophosphate dehydrogenase blockers (mycophenolate acid).

Clinical and laboratory data were retrieved through review of electronic medical records and included the following: recipient age; sex; anthropometric measurements; cause of end-stage kidney disease; donor type (ABO-compatible living donor transplant, ABO-incompatible living donor transplant, deceased donor transplant); total number of human leukocyte antigen (HLA) mismatches.

### 4.3. Outcome Variables

We determined MMP-9 transcripts in mononuclear cells eight days after transplant in kidney transplant recipients who received induced therapies with Basiliximab, Thymoglobulin, and Rituximab. We also investigated allograft function, i.e., plasma creatinine.

Delayed graft function (DGF) was defined according to the United Network for Organ Sharing as dialysis within first week after transplantation [23,24,25]. Need for dialysis was considered by the treating physicians according to local guidelines and best medical care after transplantation. Need for dialysis within the first week after transplantation was confirmed with chart review.

### 4.4. Isolation of Peripheral Blood Mononuclear Cells

Blood samples were taken eight days after incident kidney transplantation. We used the protocol for isolation of peripheral blood mononuclear cells (PBMCs) as previously described [26,27,28]. In short, PBMCs were isolated from heparinized whole blood. Blood samples were centrifuged for 4 min at 1620× *g*, and supernatant was removed. Blood was diluted with 1500 μL Hank’s Balanced Salt Solution (HBSS), carefully layered on 1800 μL Histopaque (Sigma Aldrich; Søborg, Denmark; density 1.077g/mL), and centrifuged for 15 min at 952× *g*. PBMCs are extracted from the interphase and washed in phosphate-buffered saline via centrifugation for 4 min at 8000× *g*. The supernatant was decanted. The pellet was suspended in 400 μL TRI reagent (TRIzol, Sigma Aldrich, Søborg, Denmark) and stored at −80 °C.

### 4.5. Purification of Total RNA and Synthesis of Complementary DNA

We used the protocol as previously described [26,27,28]. Briefly, we used a RNeasy mini kit including RNAse-free DNase set (Qiagen, Hilden, Germany) according to the manufacturer’s protocol. Concentrations and purity of total RNA were assessed using a UV-visible spectrophotometer (Implen nanophotometer, Implen, Munich, Germany) with absorbance at 260 nm and 280 nm, and the ratio (A260/A280) was used to assess RNA purity. At least 1.8 indicated a highly purified RNA sample, which was included for further processing.

Using QuantiTect Reverse Transcription Kit (Qiagen, Hilden, Germany), the extracted RNA was reverse transcribed into cDNA. To eliminate genomic DNA, 300 ng of total RNA was incubated in gDNA wipeout buffer at 42 °C for 4 min. Reverse transcription was initialized by mixing the total RNA with Reverse transcription Master Mix (Quantiscript Reverse Transcriptase, Quantiscript RT buffer and RT primer mix), and incubated at 37 °C for 60 min. To inactivate the reaction, temperature was raised to 95 °C for 5 min and cDNA was stored at −20 °C.

### 4.6. Primers and Quantitative Real-Time Reverse Transcriptase-Polymerase Chain Reaction (qRT-PCR)

We used the protocol as previously described for other markers from our lab [26,27,28]. To quantify the amount of MMP-9 in our samples, qRT-PCR was performed. Gene-specific oligonucleotide primers were used from Sigma Aldrich with the following sequence:

MMP-9 primer sequence

5′AGACACCTCTGCCCTCACCATGAG3′;

5′GGTTCGCATGGCCTTCAGCGT3′.

β-actin primer sequence:

5′GGACTTCGAGCAAGAGATGG3′3;

5′AGCACTGTGTTGGCGTACAG3′.

**Figure 2:**
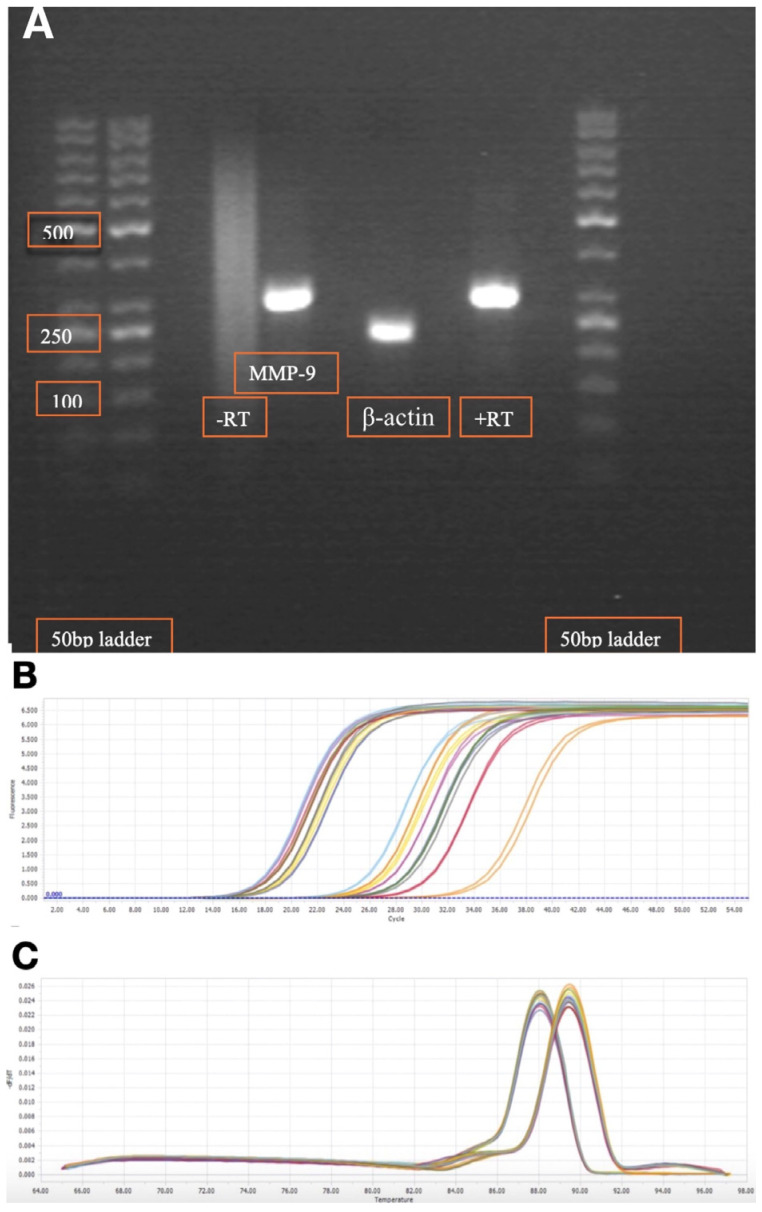
(**A**) Gel electrophoresis of matrix metalloproteinase type 9 (MMP-9) and β-actin of qRT-PCR transcripts in peripheral blood mononuclear cells. (**B**) Amplification curves. (**C**) Melting peaks. This figure shows cycles for kidney transplant recipients on day 8 post-transplantation. Higher number of cycles indicates a lower amount of target transcript.

The sequences were also checked from the current literature [28,29]. The target base pair (bp) PCR products for MMP-9 and β-actin transcripts were 307 and 234 bp, respectively. To ensure primer efficiency, electrophoresis through a 2% agarose gel was used for visual control of PCR product band size (Figure 2A).

The qRT-PCR measurements were obtained using the LightCycler 96 Instrument (Roche, Copenhagen, Denmark) according to the manufacturer’s instructions. The following cycling conditions were used: an initial preincubation cycle at 95 °C for 10 min, followed by 50 cycles of a three-step amplification; denaturation at 95 °C for 10 s, primer annealing at 60 °C for 10 s, and primer extension at 72 °C for 10 s.

Amplification was performed using the FastStart Essential DNA Green Master Mix (Roche, Copenhagen, Denmark, catalogue number: 6924204001). Each reaction well had a volume of 20 μL, consisting of 2 μL of cDNA, 4 μL of nuclease-free H_2_O, 2 μL of each primer, and 10 μL of FastStart Essential DNA Green Master Mix.

Quantification cycle (Cq) values were determined using LightCycler 96 Software 1.1 (Roche, Copenhagen, Denmark). The Cq values were obtained from amplification plots. Amplification plots and melting peaks were visually inspected to secure sample specificity and avoid impurity. Examples of amplification curves are shown in Figure 2B,C.

A nuclease-free water control was included in each PCR plate run, as well as inhouse control cDNA, and all reactions were performed in duplicate. The relative fold change in gene expression compared to health control subjects was calculated using the ΔΔCq method (2−ΔΔCq method) [30].

### 4.7. Statistical Analysis

Continuous data are presented as median and interquartile range (IQR). Frequency counts were calculated in categorical data and compared with Fisher’s exact test or chi-squared test as appropriate. Non-parametric Kruskal–Wallis test or Mann–Whitney test was performed for continuous variables, as appropriate. Data were analyzed using GraphPad prism software (version 10.4.2, GraphPad Software, La Jolla, CA, USA). All statistical tests were two-sided, with *p*-values less than 0.05 considered statistically significant.

## 5. Conclusions

Our present investigation shows increased MMP-9 transcripts in kidney transplant recipients who obtained thymoglobulin as an inflammatory response.

## Figures and Tables

**Table 1:** Clinical characteristics of all 67 kidney transplant recipients stratified according to donor status.

Characteristic	All Patients (N = 67)	AB0- Incompatible Living Donor (N = 4)	AB0- Compatible Living Donor (N = 17)	Deceased Donor (N = 46)
Age, years	55 (45.5–62.5)	54.5 (47.5–59.8)	40 (31–56)	56.5 (50.3–63.8)
Gender male, N (%)	46 (69%)	3 (75%)	9 (53%)	34 (74%)
Gender female, N (%)	21 (31%)	1 (25%)	8 (47%)	12 (26%)
Weight (kg)	80.9 (65–92.4)	78 (61.5–97.5)	74 (61.7–88)	84.2 (66.5–92.8)
Height (cm)	176 (169.5–185) a	185 (172.5–191) c	174 (171.8–182.3) d	177 (168.9–184.3) e
Body mass index (kg/m^2^)	25.4 (22.6–28.4) a	26.6 (23.3–28.3) c	24.8 (22.9–25.7) d	25.8 (22.6–28.8) e
Systolic blood pressure (mmHg)	156 (137–164)	163 (156–176)	146 (136–169)	154 (137–164)
Diastolic blood pressure (mmHg)	87 (78–95)	88 (79–103)	88 (83–103)	85 (78–94)
Delayed graft function, N (%)	7 (10%) b	0 (0%)	1 (6%)	6 (13%) i
Plasma creatinine preoperative (μmol/L)	651 (571–895) b	891 (634.3–1147.5)	795 (623–919)	640.5 (561.8–863) i
Plasma creatinine first postoperative day (μmol/L)	180 (67–441) b	393.5(326–440)	387 (306–453)	517 (392.5–712.5) i
Cause of kidney disease (%)				
Diabetic nephropathy	9 (13.4%)	0 (0%)	0 (0%)	9 (19.5%)
Hypertensive nephropathy	4 (6%)	0 (0%)	1 (5.8%)	3 (6.5%)
Glomerulonephritis	27 (40%)	2 (50%)	9 (53%)	16 (34.7%)
Polycystic kidney disease	18 (26.8%)	1 (25%)	4 (23.5%)	13 (28.3%)
Other/unknown	9 (13.4%)	1 (25%)	3 (17.6%)	5 (11%)
Induction therapy, N (%)				
Basiliximab	56 (83.6%)	3 (75%)	14 (82%)	39 (85%)
Rituximab	4 (6%)	3 (75%)	0 (0%)	1 (2.1%)
Prednisolone	22 (32.8%)	4 (100%)	10 (59%)	8 (17.4%)
Thymoglobulin	10 (15%)	1 (25%)	3 (17.6%)	6 (13%)
HLA total mismatch, range (0–6)	3 (3–4) f	3 (2–4) c	3 (2–3) g	3 (3–4) h

HLA, human leukocyte antigen. Continuous data is presented as median (IQR). Categorical data is presented as number (%). Data available a, 59 recipients; b, 65 recipients; c, 3 recipients; d, 16 recipients; e, 40 recipients; f, 56 recipients; g, 11 recipients; h, 42 recipients; i, 44 recipients.

**Table 2:** Clinical characteristics of all 67 kidney transplant recipients stratified according to induction therapy status.

Recipient Characteristics	Thymoglobulin Induction Therapy (N = 10)	All Other Induction Therapies (N = 57)	*p* Value
Age, years	56 (35.5–59)	55 (46–63)	0.4417
Gender male, N (%)	8 (80%)	38 (66.6%)	0.4869
Gender female, N (%)	2 (20%)	19 (33.3%)	0.4869
Weight (kg)	80.8 (71.7–95)	80.9 (64.8–92.2)	0.6808
Height (cm)	181 (172–185.8)	176 (169–184) b	0.4451
Body mass index (kg/m^2^)	24.8 (23.2–28.2)	25.5 (22.1–28.5) b	0.9287
Systolic blood pressure (mmHg)	143 (127–161)	157 (138–165)	0.2370
Diastolic blood pressure (mmHg)	84 (72–87)	87 (79–96)	0.2405
Delayed graft function, N (%)	1 (10%)	6 (10.5%) c	>0.999
Plasma creatinine preoperative (μmol/L)	687 (496.8–843.8)	651 (587–906) c	0.6324
Plasma creatinine first postoperative day (μmol/L)	433 (346–594) a	469.5 (352.8–639.3) d	0.5783
Cause of chronic kidney disease, N (%)			
Diabetic nephropathy	0 (0%)	9 (15.8%)	
Hypertensive nephropathy	0 (0%)	4 (7%)	
Glomerulonephritis	10 (100%)	17 (30%)	<0.01
Polycystic kidney disease	0 (0%)	18 (31.6%)	
Other/unknown	0 (0%)	9 (15.8%)	
Induction therapy, N (%)			
Basiliximab	0 (0%)	56 (98.2%)	
Rituximab	1 (10%)	3 (5.3%)	
Prednisolone	10 (100%)	12 (21%)	
Thymoglobulin	10 (100%)	0 (0%)	
HLA total mismatch, range (0–6)	3 (2–4) a	3 (3–4) e	0.9465

HLA, human leukocyte antigen. Continuous data is presented as median (IQR) and compared with Mann Whitney test. Categorical data is presented as number (%) and compared with Fisher’s exact test. Data available a, 9 recipients; b, 49 recipients; c, 55 recipients; d, 56 recipients; e, 47 recipients.

## Data Availability

All data are included in the present manuscript.

## Abbreviations

The following abbreviations are used in this manuscript:

DGF	delayed graft function
IQR	interquartile range
KTR	kidney transplant recipient
MMP-9	matrix metalloproteinase type 9
PCR	polymerase chain reaction
